# Activated Ras alters lens and corneal development through induction of distinct downstream targets

**DOI:** 10.1186/1471-213X-10-13

**Published:** 2010-01-27

**Authors:** Daniel Burgess, Yan Zhang, Ed Siefker, Ryan Vaca, Murali R Kuracha, Lixing Reneker, Paul A Overbeek, Venkatesh Govindarajan

**Affiliations:** 1 Department of Surgery, 2500 California Plaza, Creighton University, Omaha, NE 68178, USA; 2 Department of Ophthalmology, University of Missouri School of Medicine, Columbia, MO, 65212, USA; 3 Department of Molecular & Cellular Biology, Baylor College of Medicine, Houston, TX 77030, USA

## Abstract

**Background:**

Mammalian Ras genes regulate diverse cellular processes including proliferation and differentiation and are frequently mutated in human cancers. Tumor development in response to Ras activation varies between different tissues and the molecular basis for these variations are poorly understood. The murine lens and cornea have a common embryonic origin and arise from adjacent regions of the surface ectoderm. Activation of the fibroblast growth factor (FGF) signaling pathway induces the corneal epithelial cells to proliferate and the lens epithelial cells to exit the cell cycle. The molecular mechanisms that regulate the differential responses of these two related tissues have not been defined. We have generated transgenic mice that express a constitutively active version of human H-Ras in their lenses and corneas.

**Results:**

Ras transgenic lenses and corneal epithelial cells showed increased proliferation with concomitant increases in *cyclin D1 *and *D2 *expression. This initial increase in proliferation is sustained in the cornea but not in the lens epithelial cells. Coincidentally, cdk inhibitors *p27*^*Kip*1 ^and *p57*^*Kip*2 ^were upregulated in the Ras transgenic lenses but not in the corneas. Phospho-Erk1 and Erk2 levels were elevated in the lens but not in the cornea and *Spry 1 *and *Spry 2*, negative regulators of Ras-Raf-Erk signaling, were upregulated more in the corneal than in the lens epithelial cells. Both lens and corneal differentiation programs were sensitive to Ras activation. Ras transgenic embryos showed a distinctive alteration in the architecture of the lens pit. Ras activation, though sufficient for upregulation of *Prox1*, a transcription factor critical for cell cycle exit and initiation of fiber differentiation, is not sufficient for induction of terminal fiber differentiation. Expression of Keratin 12, a marker of corneal epithelial differentiation, was reduced in the Ras transgenic corneas.

**Conclusions:**

Collectively, these results suggest that Ras activation a) induces distinct sets of downstream targets in the lens and cornea resulting in distinct cellular responses and b) is sufficient for initiation but not completion of lens fiber differentiation.

## Background

Ras proteins have been shown to regulate diverse cellular processes including proliferation, migration, differentiation, apoptosis and senescence [1]. Ras proteins are small GTP-binding proteins that switch between inactive guanosine diphosphate (GDP)-bound and active guanosine triphosphate (GTP)-bound conformations [2-4]. Active GTP-bound Ras recruits Raf to the cell membrane. Raf then phosphorylates mitogen-activated and extracellular-signal regulated kinase kinases (MEKs) which in turn, phosphorylate extracellular signal-regulated kinases (Erks). Erks activate cytoplasmic substrates and translocate to the nucleus to stimulate expression of immediate early genes such as c-jun and E26 transcription factors (ETS) [1]. The Ras-Raf-Erk signaling pathway has been shown to be sufficient for transformation of murine cell lines [5-7].

Activating somatic missense mutations in Ras genes (predominantly in codons 12, 13 and 61) have been found in a number of human cancers [8]. Specific associations have been found between the three major Ras oncogenes, H-, K- and N-Ras, and specific types of malignancies [1]. For instance, K-Ras mutations are frequent in pancreatic and colon carcinomas, H-Ras mutations in bladder cancers and N-Ras mutations in melanomas and small intestine malignancies [1]. In addition, germline mutations in Ras underlie several developmental disorders including cardio-facio cutaneous syndrome (K-Ras), Costello (H-Ras) and Noonan syndromes (K-Ras) that are characterized by sporadic tumors and skeletal, cardiac and visual abnormalities [4]. The reasons for the association of specific Ras genes with specific tumors or developmental disorders are not well understood.

Analysis of H-, K- and N-Ras null mice suggests that Ras proteins perform both unique and overlapping roles during development. H-Ras and N-Ras single and double null mice are viable, fertile and develop normally [9,10]. Of the two K-Ras isoforms, 4A and 4B, 4B but not 4A is essential for embryogenesis [11,12]. K-Ras 4B null embryos die in utero due to anemia, liver and cardiac defects [11,12]. Replacement of K-Ras coding sequences with H-Ras results in viable mice [13] suggesting that H-Ras protein can perform the functions of K-Ras in K-Ras-expressing tissues presumably by activating the same set of downstream targets. Alterations in ocular development in K-Ras null mice have not been reported.

Lens differentiation is initiated when the neuroectoderm-derived optic vesicle induces the overlying surface ectoderm to upregulate the expression of transcription factors such as Pax6 and Sox2, leading to lens placode formation [reviewed in [14]]. The lens placode then invaginates to form a distinctively shaped, symmetrical lens pit. BMP receptor mediated signaling has recently been shown to be critical for this placodal invagination [15]. The lens cells subsequently detach from the overlying ectoderm to form the lens vesicle. Lens detachment requires the transcription factors FoxE3 and Pitx3 [16-18]. During vesicle closure, the posterior cells of the lens vesicle initiate fiber cell differentiation, forming the primary fiber cells. Fiber cells exit from the cell cycle, undergo dramatic elongation, and express β- and γ-crystallins. The anterior cells of the lens remain cuboidal and retain expression of Pax6 and E-Cadherin [19,20]. The anterior-posterior polarity of the lens is maintained throughout life by coordination of lens epithelial proliferation, cell cycle exit and initiation of fiber differentiation. Upregulation of the transcription factor Prox1 at the transition zone near the equator has been shown to be essential for cell cycle exit [21]. The CDK inhibitors p27^Kip1 ^and p57^Kip2 ^are upregulated at the transition zone and are necessary for proper cell cycle exit at the outset of fiber cell differentiation [22]. An inductive signal from the neural retina is critical for initiation of lens fiber differentiation [14,23,24]. Though the identity of this inductive signal is not known, in vitro and in vivo studies suggest that this signal is an FGF or FGF-like molecule [24,25]. Expression of a secreted dominant negative version of the FGFR3 or targeted deletion of FGFR1, 2 and 3 in the lens results in the delay or loss of fiber cell differentiation [26,27]. These results suggest that FGF receptor mediated signaling is necessary for initiation of lens fiber differentiation.

After lens vesicle detachment, surface epithelial cells adjacent to the lens vesicle differentiate to form the corneal epithelium. In mice, corneal epithelial differentiation is marked by Keratin 12 (K12) expression [28]. The corneal stroma and endothelium are formed by intraocular migration of neural crest and mesodermally-derived periocular mesenchymal cells [29].

Previous studies showed that activation of the fibroblast growth factor (FGF) signaling pathway in the lens epithelial cells in transgenic mice leads to cell cycle exit and premature fiber differentiation [30,31]. In contrast, FGF signaling induces the corneal epithelial cells to first proliferate and later differentiate into lacrimal or Harderian glands [32,33]. The molecular mechanisms that regulate proliferative and differentiation responses of the cornea and lens respectively have not been defined.

Activation of Ras, a downstream effector of FGF signaling, in the lenses of transgenic mice is sufficient to induce lens epithelial hyperplasia [34]. In addition, H-, K- and N-Ras genes are expressed ubiquitously within the eye [34]. However, in previous studies, downstream targets of Ras activation in the lens were not analyzed. In addition, the promoter used to drive Ras expression in the eye was lens-specific thereby precluding analysis of the effects of Ras activation in the cornea.

In order to test whether activation of Ras in the cornea can phenocopy the effects of FGF signaling, we generated transgenic mice that express a constitutively active version of H-Ras (G12V) driven by a modified Pax6 promoter [19] This promoter is active in the lens, corneal and conjunctival epithelial cells. Our results suggest that, despite the common embryonic origin of the lens and the cornea, these two tissues possess distinct sets of downstream targets that regulate the cellular responses to Ras activation.

## Methods

### Generation of Ras transgenic mice

The human H-Ras (G12V) genomic clone, encoding a constitutively active version of H-Ras (from Dr. Michael Lieberman, Baylor College of Medicine, Houston), was digested with *Spe*I and inserted between the Pax6 promoter/enhancer and the SV40 intron and polyadenylation sequences [19]. The injection fragment was generated by *Spe*I and *Stu*I digestion and was microinjected into individual pronuclei of 1-cell stage FVB/N mouse embryos. Injected embryos were transferred into pseudopregnant ICR strain female mice. Animals were handled following the guidelines provided in US Public Health Service Policy on Humane Care and Use of Laboratory Animals. The embryos were allowed to develop to term and potential Pax6-Ras transgenic mice were identified by isolating genomic DNA from tail biopsies and screening by PCR, using primers specific for the SV40 sequences [26].

### Histological Analyses

Embryos were obtained by mating FVB/N females to heterozygous Ras transgenic males. Pregnant females were sacrificed at appropriate time points and transgenic offspring were identified by PCR. Heads of transgenic mice were removed, fixed in 10% formalin, dehydrated, embedded in paraffin, sectioned (5-7 μm) and used for histological analyses, in situ hybridization and immunohistochemistry.

### In situ hybridization

To analyze patterns of gene expression, [^35^S] UTP-labeled riboprobes were generated. The SV40 antisense riboprobe was synthesized using *EcoR*I-digested SV40 DNA and T3 RNA polymerase (Promega). The *FoxE3 *antisense probe was synthesized using *Hin*dIII-digested mouse *FoxE3 *cDNA and T3 RNA polymerase. The *Pitx3 *antisense probe was synthesized using *Xho*I-digested mouse *Pitx3 *cDNA and SP6 RNA polymerase. The *Prox1 *antisense probe was synthesized using *Sma*I-digested mouse *Prox1 *cDNA and T3 RNA polymerase. The *p57*^*Kip*2 ^antisense probe was synthesized using *Bam*HI-digested mouse *p57*^*Kip*2 ^cDNA and T3 RNA polymerase. The *Erm*, *Pea3, Spry1 *and *Spry4 *antisense probes were synthesized using *Eco*RI-digested *Erm, Pea3, Spry1 and Spry4 cDNAs *and T7 RNA polymerase. The *Spry2 *antisense probe was synthesized using *Sac*I-digested mouse *Spry2 *cDNA and T3 RNA polymerase. The *cyclin D1 *antisense probe was synthesized using *Xho*I-digested mouse *cyclin D1 *cDNA and T3 RNA polymerase. The *cyclin D2 *antisense probe was synthesized using *Sph*I-digested mouse *cyclin D2 *cDNA and T7 RNA polymerase. The *cyclin B1 *antisense probe was synthesized using *Hin*cII-digested mouse *cyclin B1 *cDNA and T7 RNA polymerase. The *p21*^*Cip*1 ^antisense probe was synthesized using *Apa*I-digested mouse *p21*^*Cip*1 ^cDNA and T7 RNA polymerase. The *Hes1 *antisense probe was synthesized using *Hin*dIII-digested mouse *Hes1 *cDNA and T7 RNA polymerase. In situ hybridizations were performed using the same hybridization and washing conditions as described previously [32]. The hybridized slides were soaked in Kodak NTB-2 emulsion, dried and exposed for 4-10 days at 4°C. Following development and fixation, the slides were counterstained with hematoxylin. Bright and dark-field images were captured separately using a Nikon Eclipse E600 microscope. Silver grains in the dark field images were pseudo-colored red using ADOBE Photoshop CS and overlaid on corresponding bright field images.

### Immunohistochemistry

Immunohistochemistry on paraffin-embedded tissue sections was performed as follows. Slides containing ocular sections were first deparaffinized and rehydrated. Antigens were retrieved by microwave treatment in 10 mM Sodium Citrate buffer (pH 6.0). Following antigen retrieval, the tissue sections were blocked with 10% normal horse serum for 30 minutes, at room temperature. The slides were then incubated with anti-Pax6 (catalog number PRB-278P, 1:250; Covance, Berkeley, CA), anti-E-Cadherin (catalog number 610181, 1:1000; BD Transduction Laboratories, San Jose, CA), anti-Keratin 14 (Krt14) (catalog number PRB-155P, 1:2000; Covance), anti-Prox1 (catalog number ab5475, 1:1000; Chemicon, Temecula, CA), anti-α A crystallin (catalog number ab5595, 1:10,000; Abcam, Cambridge, MA), anti-β crystallin (catalog number sc-22745, 1:400; Santa Cruz Biotechnology, Santa Cruz, CA), anti-Keratin 12 (Krt12) (catalog number KAL-KR074, 1:200; Cosmo Bio Company, Tokyo, Japan), anti-phospho Erk1/2 (catalog number 4370, 1:50; Cell Signaling Technology, Danvers, MA), anti-p57^Kip2 ^(catalog number ab4058, 1:250; Abcam), anti-14-3-3σ (catalog number sc-7683, 1:100; Santa Cruz Biotechnology), anti-Ki67 (catalog number M7249, 1:200, Dako Cytomation, Carpinteria, CA) or anti-Trp63 (4A4) (catalog number sc-8431, 1:100; Santa Cruz) antibody overnight at 4°C. Following brief washes in PBS, the slides were incubated with the appropriate biotinylated-secondary antibodies; anti-rabbit IgG (catalog number BA-1000, 1:200; Vector Labs, Burlingame, CA) for Pax6, Prox1, α A-crystallin, β-crystallin, phospho-Erk1/2, p57^Kip2^, Krt12 or Krt14, anti-mouse IgG (catalog number BA-2000, 1:200; Vector Labs) for E-Cadherin or Trp63, anti-goat IgG (catalog number BA-5000, 1:200; Vector Labs) for 14-3-3σ and anti-rat IgG (catalog number BA-4000, 1:200, Vector Labs) for Ki67 for 30 minutes at 37°C. Antigen-antibody complexes were then detected using streptavidin-linked Alexa 594 (Invitrogen, Carlsbad, CA) at 1:1000 dilution. Sections were mounted using ProLong Antifade media containing DAPI (Invitrogen, Carlsbad, CA). Images were captured using a Nikon Eclipse E600 microscope.

### Proliferation assay

DNA replication was examined by BrdU incorporation as described previously [26]. Cell proliferation was analyzed by counting the number of BrdU positive nuclei. Quantification of cell proliferation (BrdU labeling index) was performed by determining the fraction of BrdU labeled nuclei over the total number of nuclei present on a given section. Cells in the interior of the Pax6-Ras transgenic lenses that expressed markers such as FoxE3 and Prox1 were counted as lens cells. A minimum of 3 different embryos were analyzed per genotype/time point. The number of sections used for quantification is indicated in the graphs (Fig. 4I). Analysis was performed by two-tailed Student's t-test at P ≤ 0.05.

## Results

**Figure 1:**
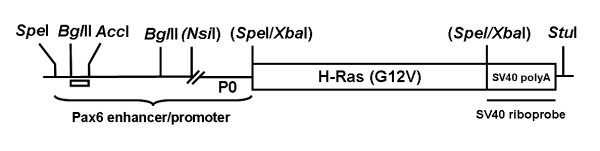
**Schematic diagram of the Pax6-Ras transgene**. A human H-Ras (G12V) genomic clone (~3.1 Kb) was inserted between a modified Pax6 promoter/enhancer (2.3 Kb) and an intron and polyadenylation (polyA) sequence derived from the SV40 virus (850 bp). The lens/cornea enhancer is contained between the *Bgl*II/*Acc*I sites (clear box). The SV40 polyA sequences were used to make riboprobes for detection of transgene expression.

In order to test whether activation of Ras, a downstream effector of FGF signaling, can mimic the effects of FGF stimulation on the lens and cornea in the murine eye, we generated transgenic mice with targeted expression of a constitutively active (and oncogenic) version of human H-Ras under the control of the Pax6 promoter (Fig. 1). This promoter is active from embryonic day 9.5 (E9.5) in the lens, corneal and conjunctival epithelial precursors [19]. The Pax6-Ras transgene was constructed by inserting the human H-Ras gene (G12V) between the Pax6 enhancer/promoter and SV40 intron and polyadenylation site. The transgene was microinjected and two founders were generated. Stable transgenic lines (OVE2043, OVE2044) were established from these founders. Transgenic line OVE2043 did not show any ocular abnormalities. OVE2044 mice showed microphthalmia and were born with open eyelids.

### Transgene expression

**Figure 2:**
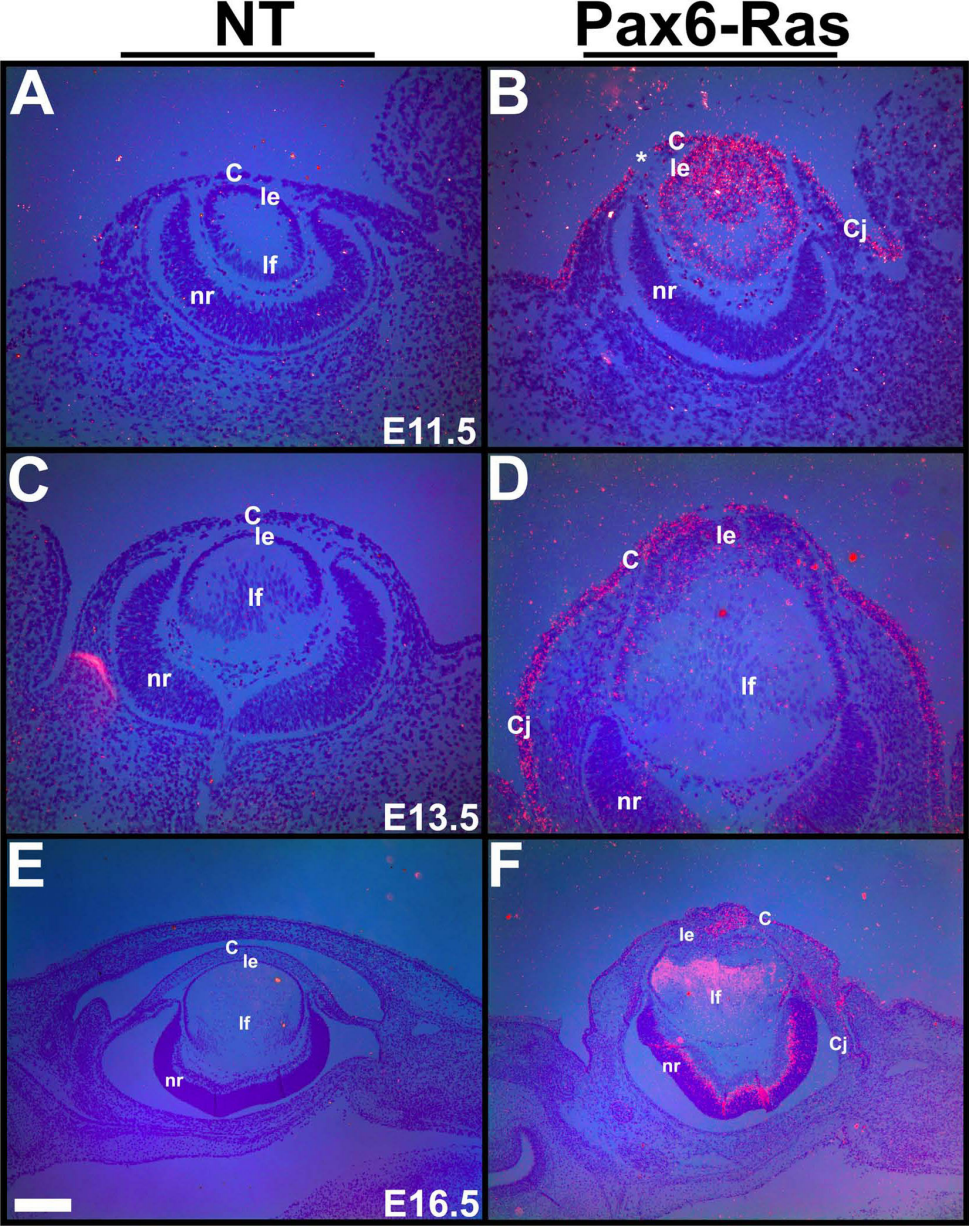
**Transgene expression**. In situ hybridizations were performed on ocular sections of nontransgenic (A, C, E) and Pax6-Ras transgenic (B, D, F) embryos using an ^35^S-labeled SV40 riboprobe. Dark-field images were overlaid on respective bright-field images and silver grains were pseudocolored red. Ras transgene expression was seen in the lens, cornea and conjunctival epithelial cells at E11.5 and E13.5 (B, D) and in some retinal neuroblasts at E13.5 and E16.5 (D, F). Discontinuity in the corneal epithelium in panel B (asterisk) is due to a sectioning artifact. Abbreviations; C, corneal epithelium; Cj, conjunctival epithelium; le, lens epithelium; lf, lens fibers; nr, neural retina. The scale bar (E) represents 100 μm in panels A-D and 250 μm in panels E, F.

Transgene expression in the two Pax6-Ras transgenic lines was examined by in situ hybridizations (Fig. 2). No transgene expression was seen in the OVE2043 line (data not shown). In the OVE2044 line, at E11.5 and E13.5, transgene expression could be seen in the lens, corneal and conjunctival epithelial cells (Fig. 2B, D). Low levels of Ras transgene expression were seen in a subset of neuroblasts in the retina at E13.5 (Fig. 2D) with stronger expression at E16.5 (Fig. 2F). As transgene expression was seen only in the OVE2044 transgenic line, further studies were performed on this line and the results are presented here. In this manuscript we study the initial changes in lens and corneal differentiation. Later changes in retinal organization are not evaluated in this report.

### Ras transgenic mice show altered lens and corneal development

**Figure 3:**
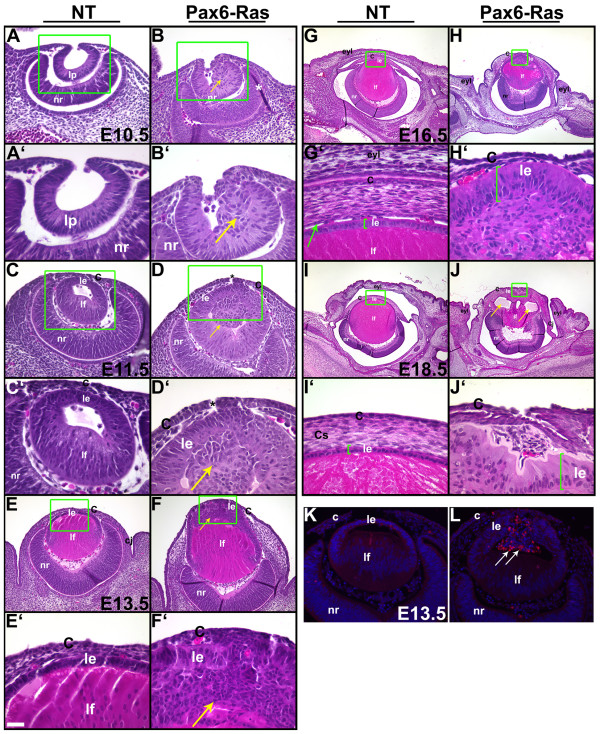
**Pax6-Ras transgenic mice show altered lens and corneal development**. Heads of E10.5 (A-B'), E11.5 (C-D'), E13.5 (E-F'), E16.5 (G-H') and E18.5 (I-J') nontransgenic (NT) and Pax6-Ras transgenic mice were sectioned and stained with hematoxylin and eosin. Panels A-J' are higher magnifications of boxed regions in A-J respectively. Asterisks in panels D, D' indicate sectioning artifacts. Arrows in B, B', D and D', point to abnormal clusters of cells in the interior of the lens. Arrow in G' points to the corneal endothelium. Arrows in J point to the vacuoles within the lens. Lens epithelial hyperplasia was seen in the Ras transgenic embryos (arrow in F') and Ras transgenic lenses failed to detach from the surface ectoderm (D, D'). Eyelids failed to close in Ras transgenic embryos (H) and the corneal stromal cells were disorganized (F, H, J). K, L. Activated caspase 3 was detected by immunohistochemistry (L, arrows). Antigen-antibody complexes are in red and the nuclei are stained blue with DAPI. Abbreviations; C, corneal epithelium; Cj, conjunctival epithelium; eyl, eyelid; le, lens epithelium; lf, lens fibers; lp, lens pit; nr, neural retina. The scale bar (E') represents 50 μm in panels A, B, C, D, K, L, 100 μm in panels E, F, 25 μm in A'-H' and 250 μm in panels G-J.

In order to assess the alterations in ocular development and morphology, sections of Pax6-Ras transgenic mice were stained with hematoxylin and eosin (Fig. 3). Nontransgenic littermates were used as controls. At E10.5, the lens placode had invaginated to form the lens pit (Fig. 3A, A'). In the Ras transgenic embryos, morphological changes at the anterior margin of the lens pit were visible (Fig. 3B, B'). The boundary between the surface and lens pit epithelial cells was well defined in the nontransgenic embryos (Fig. 3A, A'). In contrast, in the transgenic embryos, extra cells extending into the interior of the lens pit were seen (Fig. 3B, B', arrow). At E11.5, Ras transgenic lenses and corneas displayed epithelial hyperplasia (Fig. 3D, D'). In addition, the extra cells seen in the lens pit were internalized within the lumen of the developing lens. (Fig. 3D, D', arrow). By E13.5, Ras transgenic lenses had become embedded in the cornea (Fig. 3F, F'). At E16.5, the eyelids had failed to close in the Ras transgenic embryos (Fig. 3H). In addition, lenses remained attached to the cornea and the lens epithelial cells showed an elongated morphology (Fig. 3H', bracket) in contrast to the cuboidal morphology in the lens epithelial cells of nontransgenic embryos (Fig. 3G', bracket). The corneal stroma was disorganized and a distinctive corneal endothelium was not seen (Fig. 3H'). At E18.5, vacuoles were present inside the defective lens (Fig. 3J, arrows) and the central cornea showed epithelial and stromal defects (Fig. 3J, J').

### Increased apoptosis in Ras transgenic lenses

In order to assess apoptosis in Ras transgenic lens and corneal epithelial cells, we examined cleavage of caspase-3, a critical mediator of apoptosis, by immunohistochemistry (Fig. 3K, L). Activated caspase 3 was detected in the transgenic epithelial cells within the lens that were not in contact with the basement membrane (Fig. 3L, arrows). Positive staining was not seen in the lens epithelial cells that expressed the Ras transgene but retained contact with the basement membrane. Similarly, activated caspase 3 staining, for the most part, was not seen in the Ras transgenic corneas (Fig. 3L).

### Increase in proliferation of Ras transgenic lens and corneal epithelial cells

**Figure 4:**
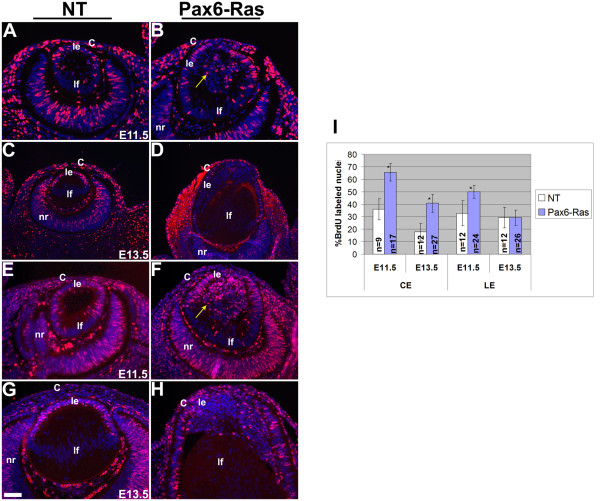
**Cell proliferation in Ras transgenic mice**. BrdU (A-D) and Ki67 (E-H) immunohistochemistry was performed on nontransgenic (NT) (A, C, E, G) and Pax6-Ras transgenic (B, D, F, H) embryos of age E11.5 (A, B, E, F) and E13.5 (C, D, G, H). Antigen antibody complexes are in red and nuclei are stained blue with DAPI. Quantification of the BrdU proliferation index is shown in panel I. Ras transgenic lens epithelial cells showed a significant increase in BrdU incorporation at E11.5 (I) but not at E13.5 (I). The number of sections counted for each genotype and time point are indicated in the graph. Error bars indicate standard deviation. Asterisk in the graph indicates a significance of P < 0.05. The scale bar (G) represents 50 μm in panels A, B, E-H and 100 μm in panels C, D.

In order to assess cell proliferation in the Ras transgenic embryos, BrdU incorporation assays and Ki67 immunohistochemistry were performed (Fig. 4). The BrdU incorporation assay allows the identification of cells in S phase of the cell cycle. Ki67 is expressed by cycling cells in G1, S, G2 and M phase but not G0 cells. Ras transgenic lenticular and corneal epithelial cells show a significant increase in BrdU labeling at E11.5 (Fig. 4B, I). The cells in the interior part of the lens vesicle that were not in contact with the basement membrane also incorporated BrdU (Fig. 4B, arrow). At E13.5, a significant increase in the BrdU labeling index of corneal, but not lens, epithelial cells was seen (Fig. 4D, I). Both BrdU (Fig. 4A-D) and Ki67 (Fig. 4E-H) assays showed consistent results. Thus, Ras transgenic lenticular and corneal epithelial cells show a significant increase in proliferation initially but later, this increase is sustained in the corneal but not in the lens epithelial cells. Alterations in conjunctival epithelial proliferation will be described elsewhere (DB, ES, RW, VG).

### Altered lens differentiation in Ras transgenic mice

**Figure 5:**
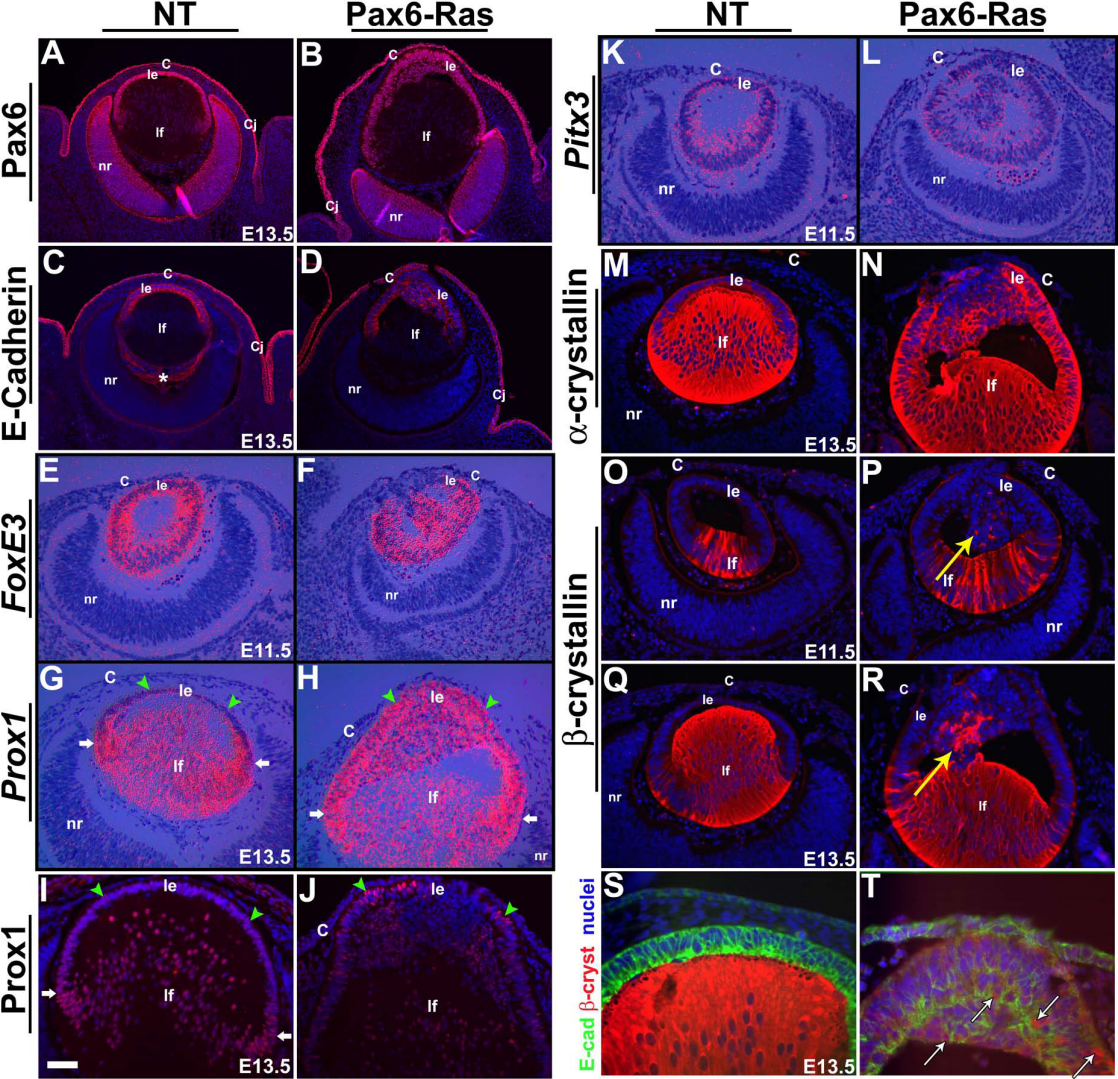
**Altered lens differentiation in Ras transgenic embryos**. Immunohistochemistry (A-D, I, J, M-T) and in situ hybridizations (E-H, K, L) were performed on nontransgenic (NT) and Pax6-Ras transgenic embryos to detect expression of Pax6 (A, B), E-Cadherin (C, D, S, T), *FoxE3 *(E, F), *Prox1 *(G-J), *Pitx3 *(K, L), α-crystallin (M, N) and β-crystallin (O-T). In panels A-D, I, J and M-R, antigen antibody complexes are in red and in panels S and T, green and red. In all these panels nuclei are stained blue with DAPI. In situ hybridizations were performed using ^35^S-labeled riboprobes (E-H, K, L). Dark-field images were overlaid on respective bright-field images and silver grains were pseudocolored red. Upregulation of *Prox1 *(H, J) expression in a subset of Ras transgenic lens epithelial cells suggests initiation of early fiber differentiation. Co-localization of β-crystallin and E-Cadherin was seen in some of the Ras transgenic lens cells (T, arrows). The staining in the vitreous (panel C, asterisk) is due to anti-mouse secondary antibody binding to IgGs in the blood vessels. White arrows in panel G, H and I point to the lens equatorial region where fiber differentiation is initiated. Green arrowheads point to lens epithelial cells (G-J). Abbreviations; C, corneal epithelium; Cj, conjunctival epithelium; le, lens epithelium; lf, lens fibers; nr, neural retina. The scale bar (I) represents 100 μm in panels A-D, 25 μm in panels I, J, S, T and 50 μm in panels E-H, K-R.

Alterations in the lens differentiation program were assessed by in situ hybridization and immunohistochemistry (Fig. 5). Pax6, FoxE3, Prox1 and Pitx3 are transcription factors that are critical for lens specification (Pax6), lens vesicle detachment (FoxE3, Pitx3) and cell cycle exit of lens epithelial cells (Prox1) [16-18,21,35-37]. In nontransgenic lenses, Pax6, E-Cadherin and *FoxE3 *are expressed in the epithelial cells (Fig. 5A, C, E), β-crystallin in differentiated fiber cells (Fig. 5O, Q) and Prox1 and α-crystallin in both epithelial and fiber cells (Fig. 5G, I, M). Expression of Pax6 (Fig. 5B), *FoxE3 *(Fig. 5F) and *Pitx3 *(Fig. 5L) in the transgenic lens epithelial cells was similar to nontransgenic lenses (Fig. 5A, E, K). E-Cadherin expression in the transgenic lens epithelial cells was comparable, although perhaps not identical, to nontransgenic lenses (Fig. 5D, T). *Prox1 *expression is normally upregulated near the equator of the lens where the fiber differentiation is initiated (Fig. 5G, I, white arrows). In contrast, *Prox1 *was upregulated in the Ras transgenic lens epithelial cells (Fig. 5H, J, arrowheads) but expression in the fiber cells was similar to wild type controls (Fig. 5H, J). Expression of α-crystallin in the transgenic lens epithelial cells (Fig. 5N) and β-crystallins in the transgenic lens fiber cells were similar to nontransgenic controls (Fig. 5O-R). The extra cells in the interior part of the Ras transgenic lenses that were not in contact with the basement membrane expressed lens epithelial markers including *FoxE3 *(Fig. 5F), *Pitx3 *(Fig. 5L) and α-crystallin (Fig. 5N). In addition, in a subset of these cells, expression of *Prox1 *(Fig. 5H, J) and β-crystallin (Fig. 5P, R, arrows) could be detected. Double labeling with β-crystallin and E-Cadherin antibodies suggested co-localization of these two proteins in some of the abnormal epithelial cells (Fig. 5S, T). These results show that some markers of lens fiber differentiation are induced in the anterior cells of the transgenic lenses.

### Erk activation in Ras transgenic lenses and corneas

**Figure 6:**
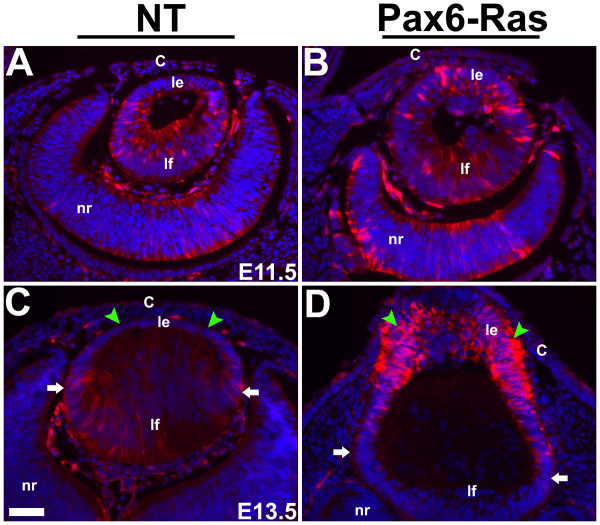
**Erk1/2 phosphorylation in Ras transgenic eyes**. Immunohistochemistry was performed on E11.5 (A, B) and E13.5 (C, D) Ras transgenic (B, D) and nontransgenic (NT) (A, C) embryos using an anti-phospho-Erk1/2 antibody. Antigen antibody complexes are in red and nuclei are stained blue with DAPI. Elevated phospho-Erk1/2 levels were detected in the Ras transgenic lens epithelial cells (Compare C and D, green arrowheads). White arrows in C and D point to the lens equatorial region where fiber differentiation is initiated. Abbreviations; C, corneal epithelium; le, lens epithelium; lf, lens fibers; nr, neural retina. The scale bar (C) represents 50 μm in panels A-D.

Erk activation in the Ras transgenic lenses and corneas was assessed by immunohistochemistry using an anti-phospho Erk1/2 antibody (Fig. 6). Erk1 and 2 are phosphorylated in response to Ras activation [38,39]. Phospho-Erk1/2 immunoreactivity could be detected in the lens fiber cells of E11.5 and E13.5 nontransgenic embryos (Fig. 6A, C). Elevated levels of phospho-Erk1/2 were detected in the Ras transgenic lens but not in the corneal epithelial cells (Fig. 6B, D). In addition, a modest reduction of phospho-Erk1/2 levels was seen in the Ras transgenic lens fiber cells at E13.5 (Fig. 6D, lf). These results show that the lens and the corneal epithelial cells show differential Erk1/2 phosphorylation in response to Ras activation.

### Ras targets in the lens and cornea

**Figure 7:**
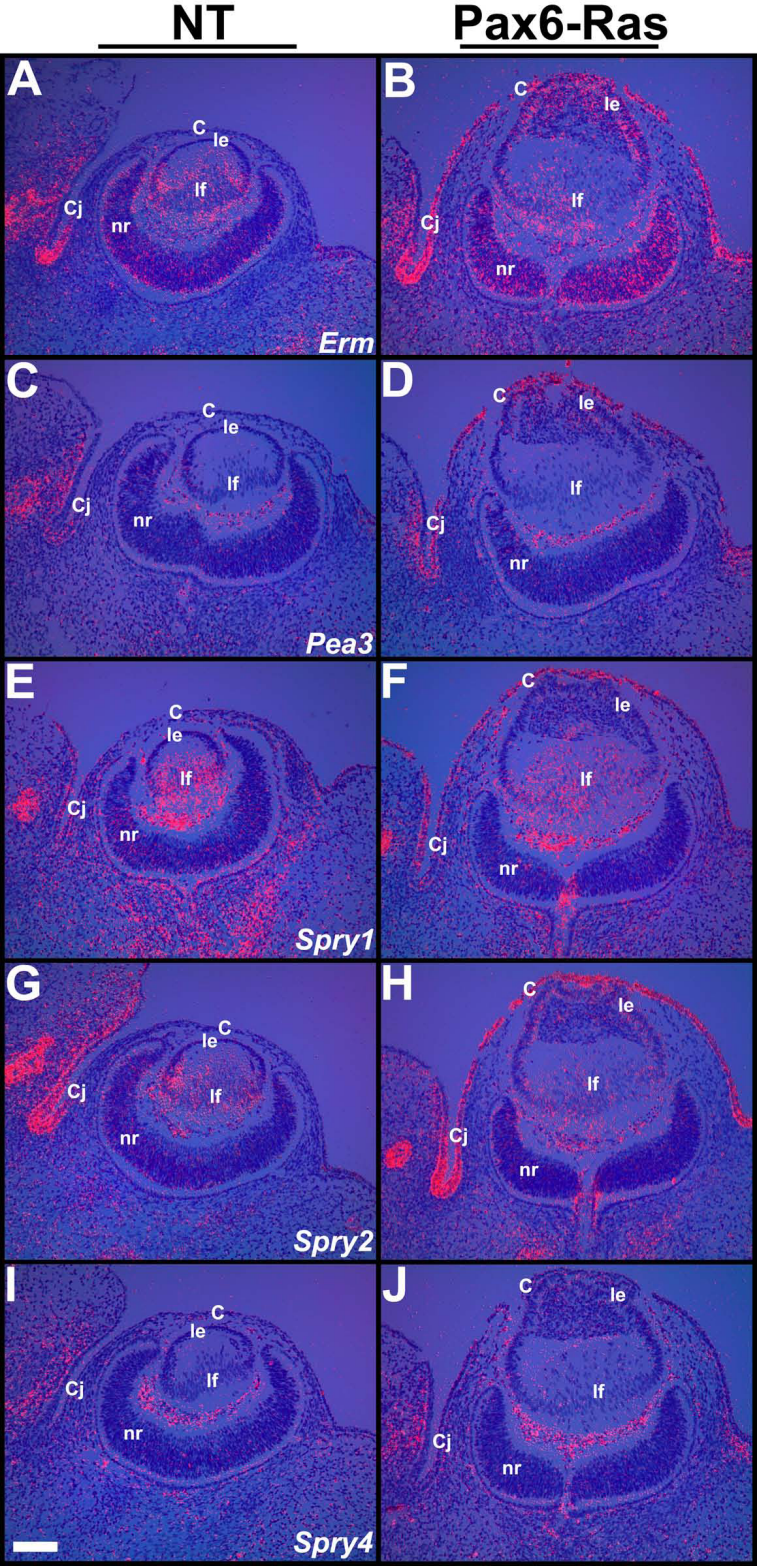
**Ras targets in the lens and cornea**. In situ hybridizations were performed on ocular sections of nontransgenic (NT) (A, C, E, G, I) and Pax6-Ras transgenic (B, D, F, H, J) E13.5 embryos using ^35^S-labelled *Erm*, *Pea3*, *Spry1*, *2 *and *4 *riboprobes. Dark-field images were overlaid on respective bright-field images and silver grains were colored red. *Erm *(B) expression was upregulated in the Ras transgenic lens and cornea. *Pea3 *(D), *Spry1 *(F) and *Spry2 *(H) expression was upregulated in the Ras transgenic corneas. Abbreviations; C, corneal epithelium; Cj, conjunctival epithelium; le, lens epithelium; lf, lens fibers; nr, neural retina. The scale bar (I) represents 100 μm in panels A-J.

In order to test whether Ras activation can induce expression of ETS transcription factors (*Erm *and *Pea3*) or negative feedback regulators of Ras-Raf-Erk signaling (*Spry1*, *2 *and *4*), in the lens and corneas, in situ hybridizations were performed on E13.5 ocular sections of Ras transgenic embryos (Fig. 7). *Erm*, *Pea3*, *Spry1 *and *2 *were upregulated in the Ras transgenic corneal and conjunctival epithelial cells (Fig. 7B, D, F, H) in contrast to nontransgenic corneas (Fig. 7A, C, E, G). Significant upregulation of *Erm*, but not *Pea3*, *Spry1 *or *2*, expression was seen in the Ras transgenic lens epithelial cells (Fig. 7B, D, F, H). *Spry 4 *was not induced in the transgenic lens or cornea (Fig. 7J). Expression of *Erm*, *Spry1 *and *2 *in the Ras transgenic lens fiber cells was similar to nontransgenic controls (Fig. 7B, F, H). In summary, different sets of downstream targets were activated in the Ras transgenic lens and corneal epithelial cells.

### Cell cycle targets of Ras in the lens and cornea

**Figure 8:**
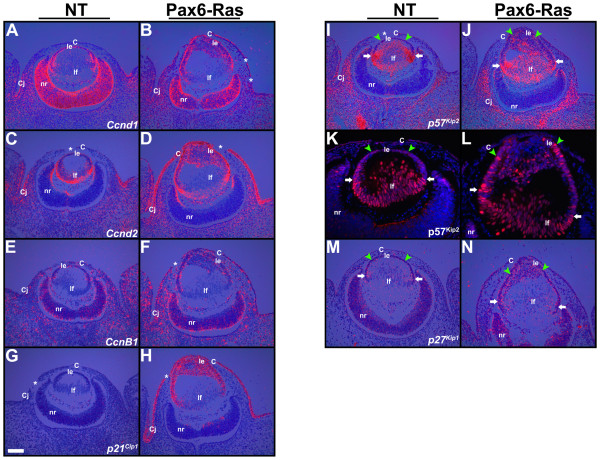
**Cell cycle targets in the lens and cornea**. In situ hybridizations (A-J, M, N) and immunohistochemistry (K, L) were performed on nontransgenic (NT) and Pax6-Ras transgenic E13.5 embryos to detect expression of *cyclin D1 *(*Ccnd1*) (A, B), *cyclin D2 *(*Ccnd2*) (C, D), *cyclin B1 *(*CcnB1*) (E, F) and cdk inhibitors *p21*^*Cip*1 ^(G, H), *p57*^*Kip*2 ^(I-L) and *p27*^*Kip*1 ^(M, N). For in situ hybridizations (A-J, M, N), dark-field images were overlaid on respective bright-field images and silver grains were colored red. For immunohistochemistry (K, L), antigen antibody complexes are in red and nuclei are stained blue with DAPI. Discontinuities in the corneal epithelium (B, C, D, F, G, H, I, asterisks) are sectioning artifacts. *Cyclins D1 *(B), *D2 *(D) and *B1 *(F) were upregulated in the Ras transgenic corneas. *p57*^*Kip*2 ^(J, L) and *p27*^*Kip*1 ^(N) were upregulated in the Ras transgenic lens epithelial cells but not corneas. *p21*^*Cip*1 ^was upregulated in the Ras transgenic lenses and corneas. Abbreviations; C, corneal epithelium; Cj, conjunctival epithelium; le, lens epithelium; lf, lens fibers; nr, neural retina. The scale bar (G) represents 100 μm in panels A-J, M, N and 50 μm in panels K, L.

Expression of genes that regulate the cell cycle was analyzed by in situ hybridization and immunohistochemistry (Fig. 8). D-type cyclins are often activated in response to growth factor and/or Ras stimulation and can promote entry into S phase [40]. *p27*^*Kip*1 ^and *p57*^*Kip*2 ^have been shown to regulate cell cycle exit during fiber cell induction [22]. Upregulation of *cyclins D1 *and *D2 *was seen in the Ras lens and corneal epithelial cells (Fig. 8B, D). *Cyclin B1 *was upregulated in the transgenic corneal epithelial cells (Fig. 8F) but expression in the lenses was similar to nontransgenic controls (Fig. 8E). No alterations in *cyclin E *expression were seen in Ras transgenic lenses and corneas (data not shown). *p21*^*Cip*1 ^expression was upregulated in both the lens and corneal epithelial cells (Fig. 8H). Expression of *p57*^*Kip*2 ^is normally upregulated near the equator where the lens epithelial cells exit the cell cycle and initiate fiber differentiation (Fig. 8I, K, white arrows). *p57*^*Kip*2 ^mRNA and p57^Kip2 ^protein levels were upregulated in Ras transgenic lens epithelial cells (Fig. 8J, L, arrowheads). Similarly, *p27*^*Kip*1 ^mRNA levels were elevated in the transgenic lens but not corneal epithelial cells (Fig. 8M, N). In summary, D-type cyclins were elevated in both the lens and corneal epithelial cells but B-type cyclins were elevated only in the corneal epithelial cells. CDK inhibitors *p27*^*Kip*1 ^and *p57*^*Kip*2 ^were preferentially upregulated in the lens but not in the corneal epithelial cells. In contrast, *p21*^*Cip*1 ^was upregulated in both epithelial cell types.

### Altered corneal differentiation in Ras transgenic mice

**Figure 9:**
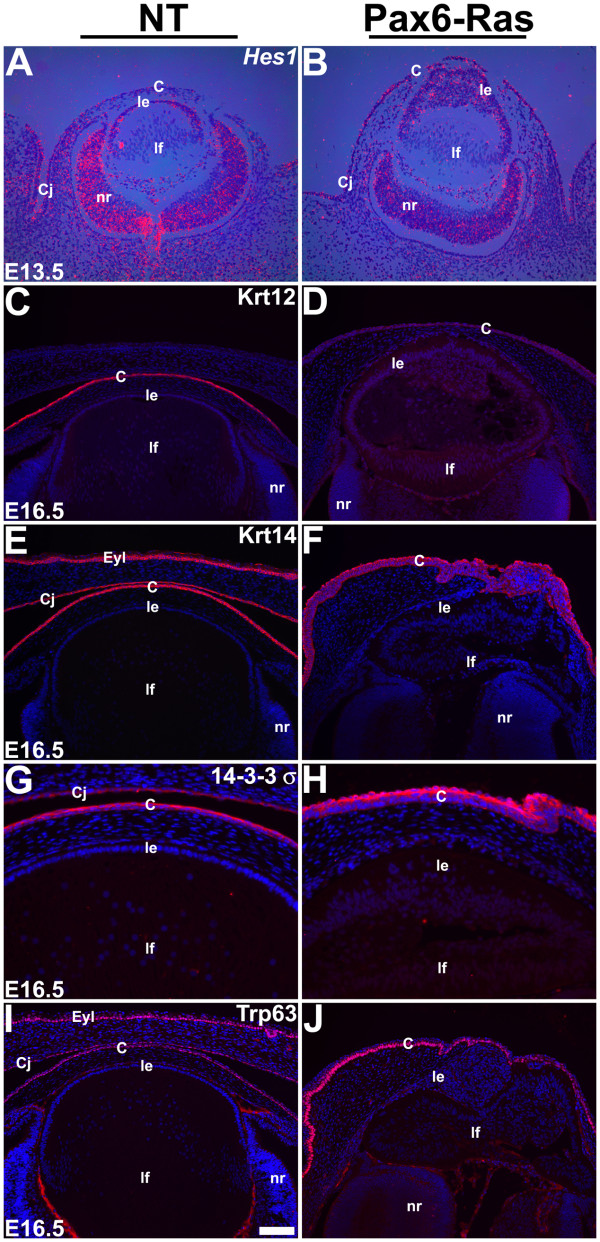
**Corneal differentiation in Ras transgenic mice**. In situ hybridizations (A, B) and immunohistochemistry (C-J) were performed on nontransgenic (NT) and Pax6-Ras transgenic embryos to detect expression of Hes1 (A, B), Keratin 12 (Krt12) (C, D), Keratin 14 (Krt14) (E, F), 14-3-3σ (G, H) and Trp63 (I, J). For in situ hybridizations (A, B), dark-field images were overlaid on respective bright-field images and silver grains were colored red. For immunohistochemistry (C-J), antigen antibody complexes are in red and nuclei are stained blue with DAPI. Ras transgenic corneal epithelial cells show an expansion of the Hes1 expression domain (B) and reduced Krt12 expression (D). Abbreviations; C, corneal epithelium; Cj, conjunctival epithelium; le, lens epithelium; lf, lens fibers; nr, neural retina. The scale bar (I) represents 100 μm in panels A-F, I, J and 50 μm in panels G, H.

Alterations in corneal differentiation in the Ras transgenic corneas were assessed by in situ hybridization and immunohistochemistry (Fig. 9). Hes1, a transcription factor and a downstream target of the Notch signaling pathway, is modestly expressed in the corneal epithelial cells (Fig. 9A) and has been shown to be critical for maintenance of the corneal progenitor/stem cells [41]. Keratin 12 (Krt12) expression is restricted to the corneal epithelial cells within the eye (Fig. 9C) and has been shown to be essential for corneal differentiation [42]. Krt14, 14-3-3σ and Trp63 are expressed in the corneal, conjunctival and skin epithelia (Fig. 9E, G, I). At E13.5, *Hes1 *expression was expanded in the Ras transgenic corneas but was unaltered in the Ras transgenic lenses (Fig. 9A, B). At E16.5, Krt12 expression was significantly reduced in the Ras transgenic corneas (Fig. 9D) but Krt14, 14-3-3σ and Trp63 were still expressed (Fig. 9F, H, J). Reduction in Krt12 expression indicates that terminal differentiation of corneal epithelial cells is inhibited by expression of activated Ras.

## Discussion

The Pax6-Ras transgenic mice were generated in order to test whether Ras activation in the corneal and lens epithelial cells can phenocopy the effects of FGF stimulation. Similar studies performed on embryonic retinal pigmented epithelial (RPE) cells in transgenic mice suggest that Ras activation can mimic the effects of FGF stimulation and is sufficient to induce the RPE cells to initiate a neuronal program of differentiation [43]. Lens-specific expression of FGF10 in transgenic mice stimulates corneal epithelial cells to first proliferate and later differentiate into lacrimal and Harderian glands. Our results suggest that Ras activation is sufficient to induce proliferation but not glandular differentiation of corneal epithelial cells. Ras transgenic lenticular and corneal epithelial cells show increased proliferation with concomitant increases in *cyclin D1 *and *D2 *expression. The cell cycle inhibitor *p21*^*Cip*1 ^was upregulated in both the lens and corneal epithelia. The initial increase in lens epithelial proliferation was not sustained perhaps due to the upregulation of *p27*^*kip*1 ^and *p57*^*kip*2^. Phospho-Erk1 and Erk2 levels are elevated in the lens but not in the cornea. Consistent with this result, expression of *Sprys1 *and *2*, downstream targets of FGF signaling and negative feedback regulators of Ras-Raf-Erk pathway, was higher in the Ras transgenic corneal than in the lens epithelial cells. *Pea3*, an ETS transcription factor, was elevated in the corneal but not in the lens epithelial cells. In contrast, transcription of *Erm*, another ETS transcription factor, was elevated in both the lens and corneal epithelial cells. Both lens and corneal differentiation programs were sensitive to Ras activation. Ras transgenic lenses showed defects in morphogenesis at the lens pit stage. Transgenic lens epithelial cells showed upregulation of Prox1, a transcription factor critical for cell cycle exit and initiation of fiber differentiation. Keratin 12, a marker of corneal epithelial differentiation, was significantly reduced in the Ras transgenic corneas. Collectively, these results show that a) lens and corneal epithelial growth and differentiation are sensitive to alterations in Ras activity and b) Ras activation can induce distinct sets of downstream targets in the lens and cornea.

In our transgenic model, the H-Ras protein is constitutively active and not subject to feedback regulation. It is therefore, possible (and perhaps likely) that Ras activity is significantly higher than normal. Upregulation of *p21*^*Cip*1 ^(a gene that is not upregulated during normal development) in the Ras transgenic lenses and corneas would be consistent with this possibility. Nonetheless, our results show that constitutive Ras signaling is not sufficient to induce glandular differentiation in the cornea. As early molecular markers of lacrimal or Harderian gland differentiation are not currently known, initiation of glandular differentiation in the Ras corneal epithelial cells could not be analyzed. Our results imply that other signaling pathways downstream of FGF receptor stimulation are necessary for ocular gland differentiation. Ras activation, however, is sufficient to induce an increase in corneal epithelial proliferation. Similarly, lens epithelial cells also respond to Ras activation by hyperproliferaton. Ras activation in the lens and corneal epithelial cells induces transcriptional upregulation of *cyclins D1 *and *D2*. As cyclin E was not induced in these tissues, it is likely that Ras-induced entry into S phase (assayed by BrdU incorporation) is due to the upregulation of cyclins D1 and D2.

Cdk inhibitors, *p27*^*Kip*1 ^and *p57*^*Kip*2^, are normally upregulated in the transition zone of the lens where the fiber differentiation program is initiated. Both *p27*^*Kip*1 ^and p57^*Kip*2 ^cooperatively regulate cell cycle exit at the onset of fiber cell differentiation [22]. Ras activation in the lenticular, but not the corneal, epithelial cells upregulated both *p27*^*Kip*1 ^and *p57*^*Kip*2^. In addition, the initial increase in proliferation seen at E11.5 in the lens epithelial cells was not sustained at E13.5 in contrast to corneal epithelial cells of the same age. These results taken together suggest that the deceleration in lens epithelial proliferation at E13.5 could be due to induction of Cdk inhibitors *p27*^*Kip*1 ^and *p57*^*Kip*2 ^in the lens. The reasons for specific upregulation of these genes in the lens but not in the cornea are not clear. However, expression of the transcription factor, Prox1, offers us a clue. The transcription factor Prox1 has been shown to be necessary for transcriptional upregulation of *p27*^*Kip*1 ^and *p57*^*Kip*2 ^in the lens as expression of these two genes is not seen in *Prox1 *null lenses [21]. In lymphatic vascular endothelial cells Prox1 expression is sufficient to activate *p57*^*Kip*2 ^expression and to promote cell cycle exit [44]. These studies considered together would suggest that transcriptional upregulation of *p27*^*Kip*1 ^and *p57*^*Kip*2 ^in Ras transgenic lenses could be due to Ras-induced upregulation of Prox1. It is not clear why Ras-induced upregulation of Prox1 is specific to the lens but not the cornea.

Ras transgenic lenses also showed increased apoptosis. Apoptosis, however, was not seen in all the lens cells that expressed the Ras transgene suggesting that apoptosis was not a non-specific consequence of Ras transgene expression. Apoptosis in the lens was seen only in the abnormal cells in the interior of the lens. These results are interesting as oncogenic Ras has been shown to block suspension induced apoptosis or anoikis in vitro [45]. Ras is able to protect cells from anoikis through the activation of Akt via PI3 kinase [45]. Whether the PI3 kinase-Akt pathway is inactive in the Ras transgenic lenses remain to be verified.

In the Ras transgenic mice, early events during lens differentiation including lens placode formation and lens invagination were not affected. However, Ras transgenic lenses failed to form a normal lens pit resulting in architectural changes at the lens pit stage. We considered the possibility that the altered lens pit architecture is caused by loss of *FoxE3 *or *Pitx3 *expression as *FoxE3 *and *Pitx3 *mutants show persistence of a lens stalk [16-18]. However, expression of both *Foxe3 *and *Pitx3 *were unaltered in Ras transgenic lenses. We also analyzed the expression of E-Cadherin, a cell adhesion protein that plays a role in lens invagination [46]. E-Cadherin was still expressed in the Ras transgenic lenses. It seems likely that the alterations in the architecture of the lens pit are a consequence of altered proliferation of cells in or around the lens placode in response to Ras stimulation. If this is true, it suggests that cell proliferation and invagination have to be precisely coordinated during lens invagination.

Previous studies have shown that activation of the FGF signaling pathway in the lens epithelial cells in vivo is sufficient for induction of fiber differentiation [30,31]. In vitro experiments using rat lens epithelial explants have shown that FGFs at low concentration, can induce proliferation and at high concentration, can induce cell cycle exit and differentiation [47]. Our results suggest that Ras activation in the lens epithelial cells induces a modest increase in cell proliferation, at least transiently. Concurrently, there is upregulation of expression of some of the early markers of lens differentiation such as *Prox1*, *p27*^*Kip*1 ^and *p57*^*Kip*2^. However, β-crystallin, a marker of terminal differentiation of lens fibers, was detected in only a small fraction of the Ras transgenic lens epithelial cells. Some of these cells also expressed lens epithelial markers including Pax6 and E-Cadherin, suggesting an abnormal or ambiguous state of differentiation. These results suggest that Ras activation is sufficient to initiate only limited aspects of the lens fiber differentiation program. These results are consistent with previous reports that show Erk signaling, while necessary for FGF-induced lens fiber elongation, is not sufficient for β-crystallin expression [48,49].

Both corneal and lens epithelial cells originate from the surface ectoderm. Interestingly, our results suggest that, in spite of their common embryonic origin, the lens and the cornea activate distinctive sets of downstream targets in response to Ras activation. Phospho-Erk levels were elevated in the Ras transgenic lens but not in the cornea. In addition, negative feedback regulators of Raf-Erk signaling such as Spry1 and Spry2 are elevated more in the cornea than in the lens epithelial cells. These observations provide correlative evidence that Spry1 and 2 may be inhibiting Raf-Erk signaling in the corneal epithelial cells. Alternatively, it is possible that signaling effectors downstream of Ras that are critical for Erk phosphorylation are expressed in the lens more than in the cornea. This model would predict that Ras-induced expression of downstream targets in the cornea is independent of Erk activation and that Ras signals through other effectors. Transcription factors that show tissue-specific expression such as Trp63 in the cornea or Prox1 in the lens may also dictate the response of these tissues to Ras signaling. Nonetheless, the differential activation of downstream targets in these two related tissues is a novel discovery that warrants further analysis.

Our results suggest that corneal differentiation is sensitive to changes in Ras activity. Interestingly, in contrast to the lens, Ras stimulation inhibits corneal epithelial differentiation. Expression of Krt12, a marker of corneal epithelial differentiation, is significantly reduced. Expansion of the expression domain of Hes1, a gene critical for maintenance of pleuripotency [41], is consistent with the block in differentiation. Expression of other proteins such as 14-3-3σ, Krt14 and Trp63 that are normally expressed in, but not restricted to, the corneal epithelium was maintained. Therefore, it is likely that the inhibition of Krt12 expression in the Ras transgenic corneas is a specific effect of Ras stimulation.

## Conclusions

Collectively, our results suggest that Ras activation induces distinct sets of downstream targets in the lens and cornea resulting in distinct cellular responses. These results support the model that responses to Ras signaling are tissue specific and dictated by the autonomous programming of the responding tissues. These results also suggest the possibility that the associations seen between mutations in specific Ras genes and specific types of malignancies could be explained at least in part, by cell-specific activation of different signaling effectors and downstream targets.

## Authors' contributions

DB and YZ performed the histological analyses, in situ hybridizations and immunohistochemical assays to characterize the Ras transgenic mice. ES and RV synthesized riboprobes for in situ hybridization studies, harvested embryos, designed primers for construction of cDNA templates for riboprobes and participated in histological analyses. MRK performed active caspase 3 immunohistochemistry. LR helped with the design of the transgenic construct and provided critical comments on the manuscript. PAO originated the idea to generate the Pax6-Ras transgenic mice and edited the manuscript. VG designed the experiments, coordinated the studies and drafted the manuscript. All authors read and approved the final manuscript.
